# The Maize WRKY Transcription Factor ZmWRKY4 Confers Lead Tolerance by Regulating *ZmCAT1* Expression

**DOI:** 10.3390/plants15030394

**Published:** 2026-01-28

**Authors:** Long Wang, Meiying Liu, Wenfei Bi, Su Li, Chang Chen, Yang Jing, Xiong Zhang, Tong Han

**Affiliations:** 1 College of Advanced Agricultural Sciences, Weifang University, Weifang 261061, China; 20210013@wfu.edu.cn (L.W.); liumeiying0919@163.com (M.L.); bwf1325533@163.com (W.B.); ls17386546434@163.com (S.L.); 1064963455@163.com (C.C.); jingyang313@163.com (Y.J.); 2 Oil Crops Research Institute, Chinese Academy of Agricultural Sciences, Wuhan 430062, China

**Keywords:** ZmWRKY4, *ZmCAT1*, Pb stress, maize

## Abstract

Lead (Pb) severely impairs plant growth, yet the role of WRKY transcription factors in Pb tolerance in maize remains largely unknown. Here, we identified a Pb-responsive WRKY transcription factor, ZmWRKY4, whose transcript levels were rapidly and strongly induced in maize leaves following Pb exposure. Physiological and biochemical analyses showed that overexpression of *ZmWRKY4* substantially enhanced Pb tolerance in maize. Transgenic lines exhibited significantly lower malondialdehyde (MDA) levels and reduced electrolyte leakage than wild-type plants. In addition, *ZmWRKY4* overexpression increased catalase (CAT) activity and effectively limited H_2_O_2_ accumulation. Further analyses revealed that ZmWRKY4 positively regulates *ZmCAT1*, a key antioxidant gene involved in H_2_O_2_ scavenging, under Pb stress. Electrophoretic mobility shift assays and ChIP-qPCR collectively confirmed that ZmWRKY4 directly binds to W-box elements within the *ZmCAT1* promoter in vivo and in vitro, thereby activating its transcription. Together, these findings define a previously uncharacterized ZmWRKY4-*ZmCAT1* regulatory module that enhances antioxidant capacity and mitigates oxidative damage during Pb stress. This work provides new insights into the molecular mechanisms underlying heavy metal tolerance in maize and identifies a promising genetic target for developing Pb-resilient crop varieties.

## 1. Introduction

Industrial expansion has accelerated heavy metal accumulation in soils, creating a major ecological and environmental challenge worldwide [1]. Among these pollutants, lead (Pb) is of particular concern due to its high toxicity, chemical persistence, and extensive industrial use [2]. Pb uptake severely restricts plant growth and development and poses substantial health risks when transferred through the food chain [3]. Roots are the primary site of Pb absorption, where most Pb is retained, although a portion is transported to aerial tissues through apoplastic and symplastic pathways [4]. Under severe Pb stress, plants exhibit marked reductions in biomass, along with stomatal closure, leaf chlorosis, and chloroplast structural damage, collectively impairing photosynthetic capacity [5,6]. In addition, excessive Pb exposure triggers excessive reactive oxygen species (ROS) accumulation, disrupting antioxidant defenses and disturbing essential metabolic processes [7]. Pb was selected as the stress factor in this study because it represents one of the most prevalent and persistent soil contaminants in agricultural ecosystems, particularly in regions affected by mining, smelting, industrial emissions, and wastewater irrigation. Moreover, Pb primarily accumulates in roots and strongly interferes with redox homeostasis and cellular detoxification processes, making it an ideal model stress for investigating root-based stress perception, transcriptional regulation, and antioxidant defense mechanisms in plants.

WRKY transcription factors constitute one of the most ubiquitous and extensively studied families of plant transcriptional regulators and play essential roles in a wide range of biological processes [8]. Named for their conserved WRKY domain, these proteins are characterized by the signature WRKYGQK motif at the N-terminus, which specifically recognizes and binds W-box cis-elements in the promoters of target genes, thereby mediating stress-responsive transcriptional regulation [9,10]. Numerous studies have highlighted the importance of WRKY proteins in plant growth, development, and responses to environmental stresses and biotic stresses [11,12]. For instance, in Arabidopsis, AtWRKY33 participates in plant defense signaling and responds to a broad range of pathogen-related stimuli [13], while AtWRKY1 functions as a negative regulator of the plant response to Pst. DC3000 [14]. In *Oryza sativa*, OsWRKY72 regulates leaf angles by modulating LAZY1-dependent shoot gravitropism. OsWRKY11 promotes rice heading by directly binding to W-box elements in the promoter of the flowering-related genes *OsMADS14* and *OsMADS15* and activating their transcription [8]. In *Arabidopsis thaliana*, AtWRKY46 acts as a positive regulator of osmotic stress tolerance [15]. By contrast, OsWRKY63 in rice reduces cold resistance [16], while ZmWRKY104 in *Zea mays* enhances plant tolerance to salt stress [17]. Although substantial progress has been made in understanding WRKY functions in metal stress responses, studies specifically addressing WRKY-mediated Pb responses remain limited. For instance, overexpression of *AtWRKY13* in Arabidopsis activates *PDR8* and enhances cadmium (Cd) tolerance [18]. In maize, ZmWRKY64 mitigates Cd toxicity by positively regulating genes involved in Cd translocation and auxin transport [19], while ZmWRKY4 has been shown to be induced by cadmium stress and to be required for abscisic acid–mediated upregulation of key antioxidant enzymes in maize [20]. Similarly, in rice, OsWRKY22 promotes *OsFRDL4* expression and citrate secretion under aluminum (Al) stress, thereby enhancing Al tolerance [21]. Nevertheless, systematic investigations of WRKY-mediated regulatory mechanisms under Pb stress remain limited.

Maize is a major cereal crop worldwide, serving as both a staple food and an essential feed resource. However, Pb contamination is widespread in many production regions, where it not only reduces grain yield and quality but also poses a major obstacle to the sustainable development of the maize industry [1]. Recent studies have reported several maize genes involved in Pb responses. For example, overexpression of *ZmPIP2;5* enhances Pb tolerance in maize seedlings [22], while ZmbZIP54 promotes Pb^2+^ retention in the cell wall and intercellular spaces, thereby limiting cellular Pb entry and mitigating cytotoxic damage [23]. In contrast, the protein kinase ZmAKINβγ1 increases Pb accumulation by modulating pectin metabolism and nitrogen-responsive pathways [24]. Moreover, ZmbZIP54 cooperates with ZmFdx5 to regulate *ZmPRP1* expression during maize responses to Pb stress [25]. A recent study showed that ZIP54 regulated the target genes *ZmZIFL1* and *ZmNRT1/PTR*, thereby affecting metal transport and stress responses and modulating Pb tolerance in maize [23]. Despite these findings, additional Pb-responsive genes remain to be identified to fully elucidate maize Pb tolerance mechanisms.

In this study, we systematically identified and functionally characterized a Pb-responsive WRKY transcription factor, *ZmWRKY4*, from maize. Our results demonstrate that ZmWRKY4 enhances Pb tolerance by directly binding to the *ZmCAT1* promoter, thereby activating *ZmCAT1* transcription, maintaining H_2_O_2_ homeostasis, and strengthening the plant antioxidant defense system. Collectively, these findings uncover a previously unrecognized molecular pathway through which ZmWRKY4 mediates Pb tolerance in maize and provide a solid theoretical foundation for developing Pb-resistant maize varieties via targeted molecular breeding strategies.

## 2. Results

### 2.1. ZmWRKY4 Is Induced by Pb Stress

**Figure 1:**
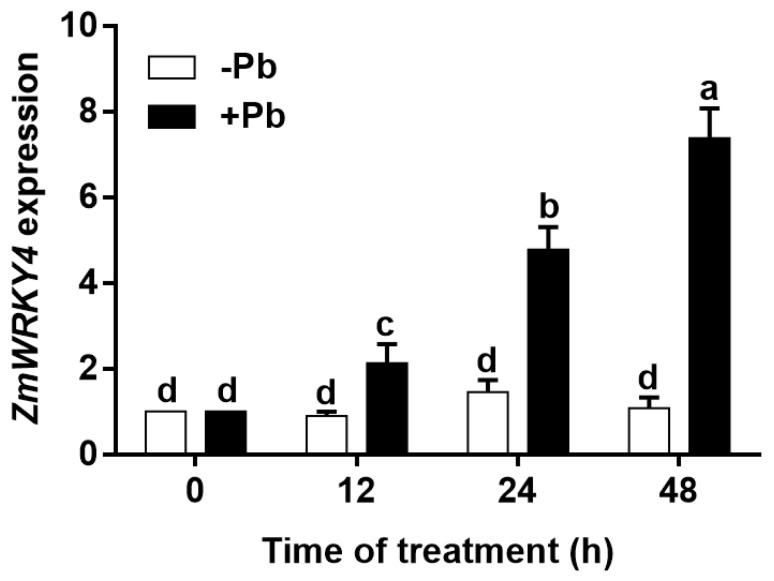
Expression pattern of *ZmWRKY4*. RT-qPCR analysis was performed to quantify *ZmWRKY4* expression in maize leaves exposed to 400 μM Pb. Data are presented as means ± standard deviation (SD) from three independent biological replicates (*n* = 3). Different letters indicate statistically significant differences among treatments at *p* < 0.05, as determined by two-way ANOVA followed by Duncan’s multiple range test.

Previous studies have shown that ZmWRKY4 is involved in cadmium-induced activation of antioxidant defense enzymes [20], prompting us to examine whether it also plays a role in the response to lead stress. Thus, 2-week-old maize seedlings (variety KN5585) were treated with 400 μM Pb(NO_3_)_2_ (designated the +Pb group), whereas seedlings grown in lead-free medium served as a control (−Pb group). Total RNA was extracted from leaves for qPCR analysis. As shown in Figure 1, under normal growth conditions (−Pb group), *ZmWRKY4* transcriptional levels remained low, indicating stable basal expression. By contrast, in the +Pb group, *ZmWRKY4* expression exhibited a time-dependent induction: it increased to approximately twofold after 12 h of Pb treatment, further rose to approximately fivefold after 24 h, and reached approximately 7.5-fold after 48 h relative to the initial level. These results demonstrate that *ZmWRKY4* is a Pb-responsive gene whose expression is specifically and strongly induced by Pb stress, suggesting that ZmWRKY4 may play a regulatory role in the maize adaptive response to Pb exposure.

### 2.2. ZmWRKY4 Positively Regulates Pb Stress Tolerance in Maize

**Figure 2:**
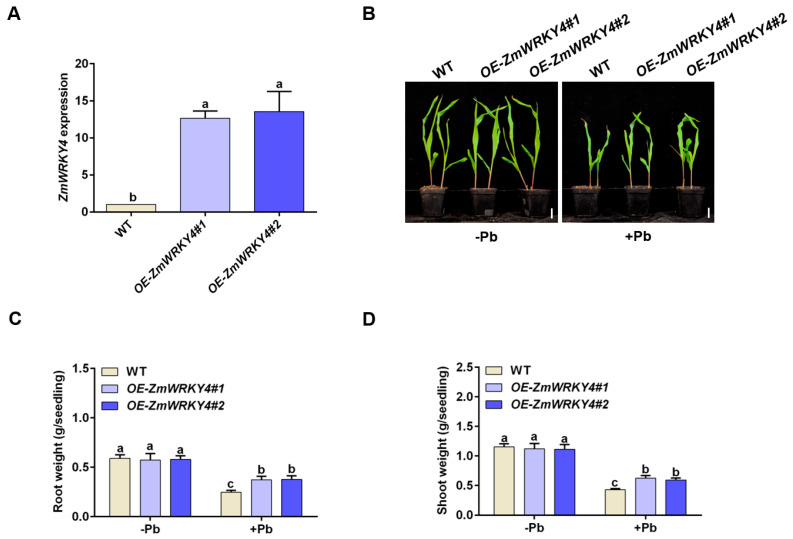
Overexpression of *ZmWRKY4* enhances maize seedling tolerance to Pb stress. (**A**) RT-qPCR analysis of *ZmWRKY4* transcript levels in overexpression lines relative to wild type (WT). (**B**) Representative images of maize seedlings grown under control conditions and after 8 d of exposure to 400 μM Pb. Scale bar = 1 cm. (**C**,**D**) Comparison of root and shoot weights of maize seedlings under normal conditions and Pb-stress conditions. Different letters indicate statistically significant differences among treatments at *p* < 0.05 according to one-way (**A**) or two-way ANOVA (**C**,**D**) followed by Duncan’s multiple range test.

To further elucidate the role of ZmWRKY4 in Pb stress responses, we generated transgenic maize plants carrying the *Ubi::ZmWRKY4* construct and obtained two independent overexpression lines (OE-*ZmWRKY4#1* and OE-*ZmWRKY4#2*). Both lines exhibited significantly higher *ZmWRKY4* transcript levels than the wild type (Figure 2A).

To assess whether ZmWRKY4 contributes to Pb tolerance, we compared the growth of *ZmWRKY4*-overexpressing plants and wild-type seedlings under Pb stress. After 8 days of Pb exposure, the overexpression lines exhibited significantly improved growth compared with the wild type (Figure 2B). Under control conditions, no phenotypic differences were observed. Consistently, both shoot and root biomass were significantly greater in the overexpression lines than in the wild type under Pb stress, whereas no differences were detected under control conditions (Figure 2C,D).

**Figure 3:**
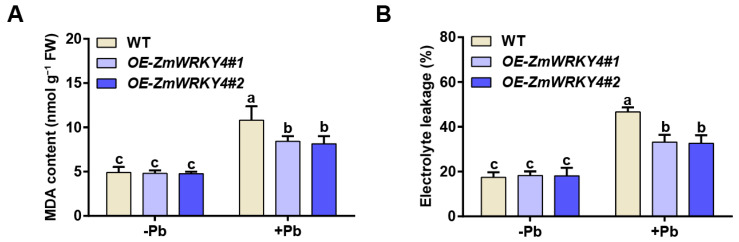
Effect of *ZmWRKY4* overexpression on oxidative damage in maize leaves under Pb stress. (**A**) Malondialdehyde (MDA) accumulation in maize leaves of *ZmWRKY4*-overexpressing lines after Pb treatment. (**B**) Electrolyte leakage in maize leaves of *ZmWRKY4*-overexpressing lines following Pb exposure. Different letters indicate statistically significant differences among treatments at *p* < 0.05 according to two-way ANOVA followed by Duncan’s multiple range test.

We further quantified malondialdehyde (MDA) content and electrolyte leakage in maize leaves. Under control conditions, both parameters were comparable between the overexpression lines and the wild type. However, following Pb treatment, the overexpression lines exhibited significantly lower MDA accumulation and reduced electrolyte leakage relative to the wild type, indicating attenuated lipid peroxidation and improved membrane integrity (Figure 3A,B). Collectively, these results demonstrate that ZmWRKY4 positively regulates Pb tolerance in maize.

### 2.3. ZmWRKY4 Enhances the Activity of CAT Under Pb Stress

**Figure 4:**
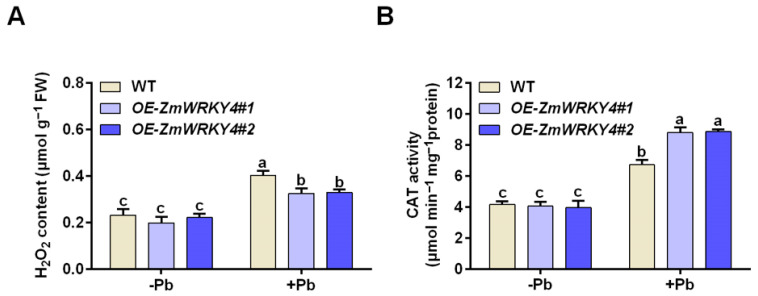
Effect of ZmWRKY4 on antioxidant enzyme activities in maize leaves exposed under Pb stress. (**A**) H_2_O_2_ content in maize leaves of *ZmWRKY4* overexpression lines following Pb exposure. (**B**) Catalase (CAT) activity in maize leaves of *ZmWRKY4* overexpression lines following Pb stress. Different letters indicate statistically significant differences among treatments at the level of *p* < 0.05 according to two-way ANOVA followed by Duncan’s multiple range test.

To evaluate the role of ZmWRKY4 in regulating oxidative stress, we measured H_2_O_2_ levels in *ZmWRKY4*-overexpressing maize lines and wild-type plants. Pb treatment markedly increased H_2_O_2_ accumulation in both genotypes; however, the overexpression lines accumulated significantly lower H_2_O_2_ than the wild type (Figure 4A). Catalase (CAT) is a key enzyme involved in H_2_O_2_ detoxification, and we therefore examined CAT activity as an indicator of oxidative stress under Pb treatment. Under Pb stress, CAT activity in the *ZmWRKY4*-overexpressing lines was 12.68% higher than in the wild type, suggesting that these plants experienced reduced oxidative stress or a more effective antioxidant response. No significant differences in CAT activity were observed between genotypes under normal conditions (Figure 4B). Together with the improved growth performance of the *ZmWRKY4*-overexpressing plants, these results indicate that ZmWRKY4 is associated with enhanced Pb tolerance and reduced oxidative damage.

### 2.4. ZmWRKY4 Positively Regulates ZmCAT1 Under Pb Stress

**Figure 5:**
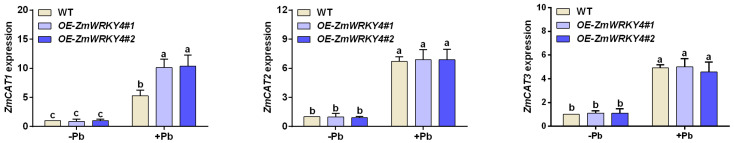
The effect of ZmWRKY4 on the *ZmCAT1* expression exposed to Pb stress. Transcription levels of catalase-encoding genes *ZmCAT1*, *ZmCAT2*, and *ZmCAT3* were measured in WT and *ZmWRKY4*-overexpressing lines. Two-week-old seedlings were treated with 0 or 400 µM Pb for 1 day. Total RNA was extracted from leaves for qPCR analysis. Different letters indicate statistically significant differences among treatments at *p* < 0.05 according to two-way ANOVA followed by Duncan’s multiple range test.

Because CAT activity was increased in the *ZmWRKY4*-overexpressing lines under Pb stress, we next examined the expression of the CAT-encoding genes *ZmCAT1*, *ZmCAT2*, and *ZmCAT3* [26]. qPCR analyses were performed on wild-type plants and *ZmWRKY4*-overexpressing lines exposed to Pb stress. *ZmCAT1* expression was markedly upregulated in the overexpression lines relative to the wild type, whereas no significant differences were observed for *ZmCAT2* and *ZmCAT3* (Figure 5). These results indicate that ZmWRKY4 may specifically regulate *ZmCAT1* expression under Pb stress.

**Figure 6:**
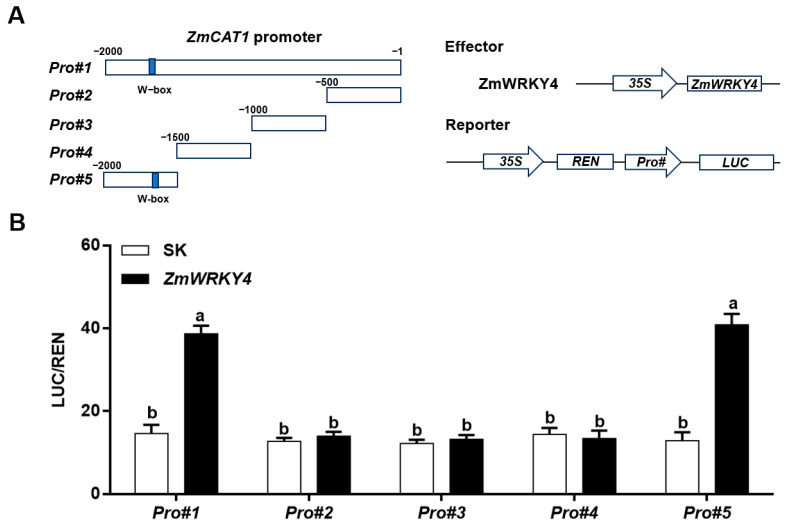
ZmWRKY4 upregulates *ZmCAT1* expression in tobacco. (**A**) Schematic diagram of promoters and reporter genes. (**B**) The *ZmCAT1* promoter, including a full-length fragment (Pro#1, −2000 to −1) and a series of truncated fragments (Pro#2, −500 to −1; Pro#3, −1000 to −500; Pro#4, −1500 to −1000; Pro#5, −2000 to −1500), were co-transformed with *ZmWRKY4* into tobacco leaves to assess their regulatory interaction. The LUC/REN ratio was used to quantify the transcriptional regulation of ZmWRKY4 on the fragments of *ZmCAT1* promoter. Different letters indicate statistically significant differences among treatments at the level of *p* < 0.05 according to one-way ANOVA.

We next examined whether ZmWRKY4 directly activates *ZmCAT1* transcription. Five fragments of *ZmCAT1* (Pro#1–Pro#5), corresponding to putative promoter regions relative to the translation start site, were amplified and fused upstream of the *LUC* reporter gene. Promoter activity was assessed using a dual-luciferase assay in *Nicotiana benthamiana* leaves. The effector plasmid contained the *ZmWRKY4* expression cassette (Figure 6A). As shown in Figure 6B, co-expression of *ZmWRKY4* with either *Pro#1* or *Pro#5* significantly increased the LUC/REN ratio compared with the empty vector (pGreen II 62-SK), whereas no activation was observed with Pro#2, Pro#3, or Pro#4. These results indicate that ZmWRKY4 directly enhances *ZmCAT1* transcription through specific promoter regions, consistent with its role in promoting ROS scavenging via upregulation *ZmCAT1*.

### 2.5. ZmWRKY4 Directly Binds to the ZmCAT1 Promoter In Vivo and In Vitro

**Figure 7:**
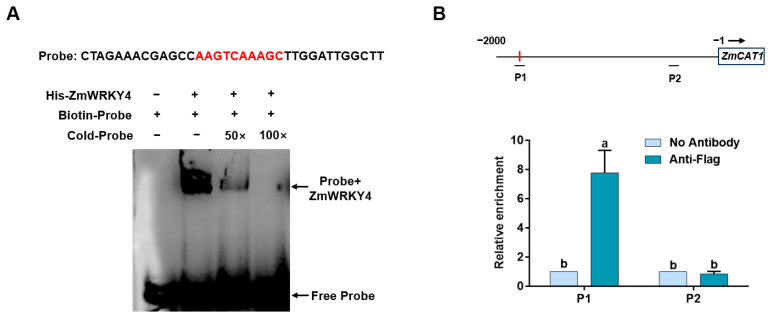
ZmWRKY4 directly binds to the *ZmCAT1* promoter in vivo and in vitro. (**A**) EMSA showed that ZmWRKY4 specifically binds to the regulatory region of *ZmCAT1*. The upper arrow indicates the ZmWRKY4–probe complex, whereas the lower arrow denotes the free probe. “Cold-Probe” refers to unlabeled probes, and “Biotin-Probe” represents labeled probes. (**B**) ChIP-qPCR analysis confirmed that ZmWRKY4 associates with the *ZmCAT1* promoter in vivo. The black horizontal lines represent the detected promoter fragments, and the red vertical lines represent the W-box. Different letters indicate statistically significant differences among treatments at the level of *p* < 0.05 according to one-way.

Given that ZmWRKY4 positively regulates *ZmCAT1* expression (Figure 5 and Figure 6), we hypothesized that ZmWRKY4 directly interacts with the *ZmCAT1* promoter. To test this hypothesis, we performed an electrophoretic mobility shift assay (EMSA) using a biotin-labeled probe containing the AAGTCAAAGC motif (−1626 to −1635 bp) from the *ZmCAT1* promoter, with unlabeled probes used as competitors. Incubation of purified His-ZmWRKY4 protein with the labeled probe produced a shifted protein–DNA complex. The shifted band was reduced by a 50-fold excess of cold competitor probe and was completely abolished by a 100-fold excess (Figure 7A). These results demonstrate that ZmWRKY4 specifically binds to the *ZmCAT1* promoter in vitro.

To verify whether ZmWRKY4 physically associates with the *ZmCAT1* promoter in vivo, we performed chromatin immunoprecipitation (ChIP)-qPCR assays using transgenic maize plants overexpressing Flag-tagged *ZmWRKY4* (*OE-ZmWRKY4#1*). Two regions of the *ZmCAT1* promoter were selected for analysis: P1, which contains the predicted AAGTCAAAGC binding motif of ZmWRKY4, and P2, which lacks this motif and served as a negative control. As shown in Figure 7B, the enrichment patterns of these promoter regions differed markedly. For the P1 region (motif-containing), the anti-Flag group exhibited a markedly high relative enrichment, approximately 8-fold higher than the background (no Antibody group). For the P2 region (motif-lacking), the anti-Flag group showed no significant enrichment, with levels comparable to the No Antibody control and the P1 background. These ChIP-qPCR results demonstrate that ZmWRKY4 specifically enriches at the motif-containing P1 region of the *ZmCAT1* promoter in vivo. These ChIP–qPCR results demonstrate that ZmWRKY4 is specifically enriched at the motif-containing P1 region of the *ZmCAT1* promoter in vivo. Together with the EMSA results confirming direct binding in vitro, these findings establish that ZmWRKY4 directly and specifically binds to the *ZmCAT1* promoter both in vivo and in vitro.

## 3. Discussion

A growing body of evidence indicates that WRKY transcription factors are key regulators of plant responses to diverse environmental stresses, including heavy metal toxicity. For example, TaWRKY70 enhances cadmium tolerance in Arabidopsis by activating *TaCAT5* [27], whereas RsWRKY75 in radish promotes ROS detoxification and cadmium efflux by upregulating *RsAPX1* and *RsPDR8* [28]. In soybean, GmWRKY21 contributes to aluminum tolerance by modulating the expression of genes responsive to acidic aluminum stress [29]. Despite these advances, the regulatory roles of WRKY proteins in maize under heavy metal stress remain poorly understood, particularly with respect to Pb exposure. In this study, we found that *ZmWRKY4* expression was induced by Pb treatment (Figure 1) and that its overexpression enhanced Pb tolerance in maize by increasing CAT activity and reducing H_2_O_2_ accumulation (Figure 3). However, the molecular mechanisms by which WRKY transcription factors regulate *CAT* expression under Pb stress remain unclear.

To counteract oxidative damage caused by heavy metal stress, plants activate antioxidant defense systems to eliminate excess reactive oxygen species (ROS) [19]. For example, transgenic Arabidopsis plants overexpressing *TaWRKY70* exhibit reduced electrolyte leakage and lower MDA and H_2_O_2_ levels compared with wild-type plants, accompanied by enhanced antioxidant enzyme activities [27]. In contrast, heterologous overexpression of *CaWRKY41* in Arabidopsis decreases cadmium tolerance, leading to greater Cd accumulation and elevated H_2_O_2_ levels [30], indicating that different WRKYs can play distinct or even opposing roles in heavy metal stress responses. WRKY transcription factors are therefore recognized as important regulators of abiotic stress tolerance through the activation of cellular antioxidant systems [12]. However, only limited information is available on the role of WRKYs in regulating antioxidant responses under heavy metal stress, particularly in crops. Previous work in maize has shown that ZmWRKY4 was induced by Cd stress and is required for the abscisic acid (ABA)-mediated upregulation of key antioxidant enzymes such as superoxide dismutase (SOD) and ascorbate peroxidase (APX). Bioinformatic analyses further revealed that the promoters of *ZmSOD4* and *ZmcAPX* contain W-box elements, suggesting that ZmWRKY4 may directly regulate their transcription [20]. Consistent with these findings, *ZmWRKY4*-overexpressing maize lines in our study displayed significantly lower MDA content and electrolyte leakage than the wild type under Pb stress (Figure 3). Moreover, these lines accumulated less H_2_O_2_ and exhibited higher CAT activity (Figure 4), supporting the conclusion that ZmWRKY4 enhances heavy metal tolerance at least in part by strengthening the antioxidant defense capacity and limiting oxidative damage.

Catalase (CAT) is a central antioxidant enzyme that decomposes H_2_O_2_ into water and oxygen, thereby maintaining cellular redox homeostasis and modulating H_2_O_2_-mediated signaling [31]. In plants, CAT enzymes are encoded by small multigene families, as reported in Arabidopsis, tobacco, maize, rice, and cucumber [26,32,33,34,35]. Numerous studies have shown that CAT genes are responsive to multiple abiotic stresses. For instance, ZmNAC84 enhances maize salt tolerance by transcriptionally activating *ZmCAT1* [26]. Heterologous expression of *TmCAT1* in yeast improves tolerance to several abiotic stresses [36]. In tobacco, *NtCATs* exhibit marked expression changes under drought conditions, highlighting their role in drought tolerance [37]. In the present study, we demonstrate that ZmWRKY4 directly binds to the *ZmCAT1* promoter and positively regulates its expression under Pb stress (Figure 6 and Figure 7). In Arabidopsis, AtCAT1 plays a major role in H_2_O_2_ detoxification under abiotic stress, whereas AtCAT2 and AtCAT3 contribute to ROS homeostasis during the light–dark cycle [38]. Overexpression of *AtCAT2* enhances tolerance to cold and drought stresses, while *AtCAT3* is mainly induced by abscisic acid and oxidative stress [39]. In maize, three CAT genes, *ZmCAT1*, *ZmCAT2*, and *ZmCAT3*, have been identified, all encoding functional CAT enzymes [26]. Consistent with their differential roles, only *ZmCAT1* expression was significantly upregulated in the *ZmWRKY4* overexpression lines following Pb exposure, whereas *ZmCAT2* and *ZmCAT3* expression remained unchanged (Figure 5).

WRKY transcription factors regulate gene expression by specifically recognizing and binding to cis-regulatory elements in target gene promoters [40]. For example, WRKY12 binds specifically to the W-box motif in the *GSH1* promoter but not in the promoters of *GSH2*, *PCS1*, or *PCS2* [41]. Similarly, WRKY13 activates *PDR8* transcription through direct promoter binding [18]. In our study, ZmWRKY4 markedly enhanced LUC activity driven by the *Pro#1* and *Pro#5* fragments of the *ZmCAT1* promoter (Figure 6), suggesting that it may directly regulate *ZmCAT1* expression. Subsequent EMSA and ChIP-qPCR analyses confirmed that ZmWRKY4 binds directly to the *ZmCAT1* promoter and positively regulates its transcription both in vitro and in vivo (Figure 7).

In summary, this study identifies *ZmWRKY4* as a Pb-responsive transcription factor in maize that enhances Pb tolerance by directly activating *ZmCAT1* expression, thereby reinforcing H_2_O_2_ scavenging and the antioxidant defense system. These findings fill a gap in understanding maize WRKY-mediated heavy metal stress regulation and provide a novel genetic target for breeding Pb-resistant maize varieties. Notably, Pb concentrations in shoots and roots were not directly quantified in this study, our phenotypic and molecular data provide indirect evidence for altered Pb uptake and translocation in maize. Pb exposure significantly inhibited root growth, indicating that roots are a primary site of Pb perception and toxicity. This observation suggests that ZmWRKY4 may influence Pb distribution and cellular handling by regulating the expression of transport- and detoxification-related genes. Future studies integrating metal content quantification with isotope tracing and cellular localization approaches will be required to directly test this hypothesis and to clarify how ZmWRKY4-mediated transcriptional regulation shapes Pb accumulation and translocation in maize. In addition, future work is also needed to explore whether ZmWRKY4 modulates additional downstream genes (beyond *ZmCAT1*) to fine-tune Pb stress responses, and to clarify its interaction with other signaling pathways in coordinating stress adaptation.

## 4. Materials and Methods

### 4.1. Plant Materials

Maize seeds (*Zea mays* L. variety KN5585) were surface-sterilized, germinated, and transplanted into small buckets containing nutrient soil. At the two-leaf stage, uniformly healthy seedlings were selected for Pb treatment. The plants were grown under controlled environmental conditions (16 h light/8 h dark; 25 °C/22 °C day/night). Pb stress was applied by a single soil drench with Pb(NO_3_)_2_ solution at a final concentration of 400 μM. After 8 days of treatment, phenotypic traits were recorded and both shoots and roots were harvested for biomass determination. The concentration of Pb(NO_3_)_2_ used in this study was selected based on preliminary dose–response experiments.

### 4.2. Generation of OE-ZmWRKY4 Transgenic Maize

Transgenic *ZmWRKY4*-overexpressing maize lines were generated in the KN5585 inbred background using an *Agrobacterium tumefaciens* strain EHA105–mediated transformation system with immature embryos as explants. The full-length coding sequence (CDS) of *ZmWRKY4* (NM_001157981.2) was amplified from maize cDNA and cloned into the expression vector under the control of the maize *ubiquitin* promoter (Table S1). The resulting construct was introduced into *Agrobacterium* EHA105 and subsequently used for transformation of KN5585 immature embryos. After co-cultivation and regeneration, putative transgenic plants were selected on medium containing Basta. Positive transformants were first identified by PCR amplification of the *ZmWRKY4* transgene, and expression levels were further examined by RT-qPCR. Independent transgenic lines exhibiting high *ZmWRKY4* expression were advanced to the T_2_/T_3_ generation and used for phenotypic, physiological, and molecular analyses. At least two independent overexpression lines were analyzed in all experiments to ensure reproducibility.

### 4.3. Real Time Quantitative PCR (RT-qPCR)

Two-week-old soil-grown maize seedlings were treated with a single soil drench of 400 μM Pb(NO_3_)_2_. Leaf samples were collected at 12 h, 24 h, and 48 h after treatment for RNA extraction. Total RNA was extracted from maize leaves using the Plant RNA Kit (Vazyme, Nanjing, China). First-strand cDNA was synthesized using the cDNA Synthesis Kit (CWBIO, Beijing, China). Relative transcript levels were calculated using the 2^−ΔΔCt^ method, with ZmActin2 serving as the internal reference gene (Table S1) [42].

### 4.4. Determination of MDA and Electrolyte Leakage

Two-week-old soil-grown maize seedlings were treated with a single soil drench of 400 μM Pb(NO_3_)_2_. Leaf samples were collected at 4 days after treatment for MDA and electrolyte leakage analysis. MDA content was determined using the thiobarbituric acid (TBA) method described by [43]. Electrolyte leakage was measured following the procedure as described in [44].

### 4.5. Determination of H_2_O_2_ Conent

Two-week-old soil-grown maize seedlings were treated with a single soil drench of 400 μM Pb(NO_3_)_2_. Leaf samples were collected at 4 days after treatment for Hydrogen peroxide (H_2_O_2_) analysis. H_2_O_2_ levels in leaves samples were quantified spectrophotometrically according to [26]. In brief, a total of 0.1 g of maize leaves was homogenized thoroughly in 1 mL of pre-chilled acetone on ice. The homogenate was transferred to an Eppendorftube, and the volume was adjusted to 1 mL with acetone to ensure consistency. The tube was centrifuged at 8000× *g* for 10 min at 4 °C in a pre-cooled centrifuge. Next, 250 μL of the supernatant was pipetted into a new EP tube as the assay tube, while a control tube was prepared by adding 250 μL of pure acetone. Subsequently, 25 μL of Reagent II and 50 μL of Reagent III were added sequentially to both tubes, and the mixtures were gently vortexed to homogeneity. After centrifugation at 4000× *g* for 10 min at 25 °C, the supernatant was discarded, and the remaining pellet was retained. Next, 250 μL of Reagent IV was added to resuspend the pellet, followed by incubation at room temperature for 5 min to ensure complete dissolution. Finally, 200 μL of the dissolved solution was transferred to a micro quartz cuvette, and the absorbance value was measured at a wavelength of 415 nm using a spectrophotometer.

### 4.6. Determination of CAT Activity

Two-week-old soil-grown maize seedlings were treated with a single soil drench of 400 μM Pb(NO_3_)_2_. Leaf samples were collected at 4 days after treatment for catalase (CAT) activity analysis. CAT activity was assessed at 240 nm using a UV spectrophotometer, following the method of [45]. In brief, 0.1 g of maize, add it to 1 mL of ice-precooled extraction buffer, and homogenize the mixture thoroughly under ice bath conditions. Transfer the homogenate to a centrifuge tube, centrifuge at 8000× *g* for 10 min at 4 °C using a pre-cooled centrifuge, and then collect the supernatant and place it on ice for subsequent assays. Then, to the sample, we added 50 μL of the supernatant and 30 μL of Reagent I, mix well, and incubated the mixture at 25 °C for exactly 10 min; subsequently, we added 100 μL of Reagent II and 265 μL of Reagent III. For the control tube: add 30 μL of Reagent I, 100 μL of Reagent II, and 265 μL of Reagent III first, mix thoroughly, and then add 50 μL of the sample supernatant. Invert both tubes gently several times to ensure complete mixing. Pipette 200 μL of the mixture from each tube into a 96-well microplate, and measure the absorbance at 405 nm using a microplate reader.

### 4.7. Dual-Luciferase Reporter Analysis

The full-length coding sequence (CDS) of *ZmWRKY4* was amplified from the cDNA of maize using gene-specific primers (Table S1), and the promoter region of *ZmCAT1* was amplified from maize genomic DNA with corresponding primers. The purified ZmWRKY4 CDS fragment was directionally cloned into the pGreen II 62-SK vector, while the *ZmCAT1* promoter fragment was inserted into the pGreen II 0800-Luc vector. Subsequently, the validated effector and reporter plasmids were co-transformed into the *Agrobacterium tumefaciens* strain GV3101 via the freeze–thaw method. For the dual-luciferase assay, the transformed *A. tumefaciens* cells were cultured to an OD_600_ of 0.8, resuspended in infiltration buffer (10 mM MES, 10 mM MgCl_2_, 150 μM acetosyringone), and incubated at room temperature for 2 h in the dark. The bacterial suspension was then injected into the abaxial side of 4-week-old *Nicotiana benthamiana* leaves. After 96 h of infiltration, leaf disks were harvested, and luciferase activities were detected using the Dual-Luciferase Reporter Gene Assay Kit (Yeasen Biotech, Shanghai, China) following the manufacturer’s instructions. The firefly luciferase (LUC) activity was normalized against the Renilla luciferase (REN) activity to eliminate the influence of bacterial infiltration efficiency [46], and the LUC/REN ratio was used to evaluate the transcriptional regulation of ZmWRKY4 on the *ZmCAT1* promoter.

### 4.8. Electrophoretic Mobility Shift Assay (EMSA)

The full-length *ZmWRKY4* coding sequence was cloned into the pET-30a vector to generate a His-tagged fusion protein and subsequently transformed into *Escherichia coli* BL21 (DE3). Transformed cells were cultured at 37 °C to an OD_600_ of ~0.6, after which protein expression was induced with 0.5 mmol L^−1^ IPTG at 30 °C for 6 h. Cells were harvested and lysed by sonication, and the recombinant His-tagged ZmWRKY4 protein was purified. Electrophoretic mobility shift assays (EMSA) were carried out using biotin-labeled DNA probes generated with the EMSA Probe Biotin Labeling Kit (Beyotime, Shanghai, China). Purified ZmWRKY4 protein was incubated with labeled probes in binding buffer for 30 min at room temperature. The protein–DNA complexes were then resolved on native polyacrylamide gels and transferred to nylon membranes. Chemiluminescent detection was performed using the Chemiluminescence EMSA Kit (Beyotime, Shanghai, China).

### 4.9. Chromatin Immunoprecipitation-Quantitative PCR (ChIP-qPCR)

The seedlings of OE-*ZmWRKY4* were harvested for chromatin immunoprecipitation (ChIP) analysis. Seedlings were vacuum-infiltrated in 1% (*v*/*v*) formaldehyde and subsequently quenched with 0.125 mol L^−1^ glycine. Chromatin was isolated and fragmented by sonication to yield DNA-protein complexes of approximately 200 bp. The sheared chromatin was incubated with an anti-Flag antibody (Abmart, Shanghai, China) to immunoprecipitate ZmWRKY4-associated DNA fragments, while an aliquot of non-immunoprecipitated chromatin was retained as input control. Following reverse crosslinking and DNA purification, the enrichment of target promoter regions was quantified by qPCR using gene-specific primers (Table S1) [47].

### 4.10. Statistical Analysis

Experimental results were presented as the mean ± standard deviation (SD) derived from no fewer than three independent repetitions. Statistical evaluations were executed using two-way analysis of variance (ANOVA) in the SPSS Statistics 20.0 software. Variations with a *p*-value < 0.05 were defined as statistically significant.

## Data Availability

The original contributions presented in this study are included in the article. Further inquiries can be directed to the corresponding authors.

## Supplementary Materials

The following supporting information can be downloaded at: https://www.mdpi.com/article/10.3390/plants15030394/s1, Figure S1: A schematic diagram of the vector used for maize genetic transformation; Table S1: The primers used in this study.

## References

[1] Zhang X., Zhao B., Ma X., Jin X., Chen S., Wang P., Zhongrong G., Wu X., Zhang H. (2024). Combining transcriptome and metabolome analyses to reveal the response of maize roots to Pb stress. Plant Physiol. Biochem..

[2] Ma L., An R., Jiang L., Zhang C., Li Z., Zou C., Yang C., Pan G., Lübberstedt T., Shen Y. (2022). Effects of ZmHIPP on lead tolerance in maize seedlings: Novel ideas for soil bioremediation. J. Hazard. Mater..

[3] Long P., Zhou X., Sang M., Li M., Li Q., Chen Z., Zou C., Ma L., Shen Y. (2024). PIP family-based association studies uncover ZmPIP1; 6 involved in Pb accumulation and water absorption in maize roots. Plant Physiol. Biochem..

[4] Kumar P.N., Dushenkov V., Motto H., Raskin I. (1995). Phytoextraction: The use of plants to remove heavy metals from soils. Environ. Sci. Technol..

[5] Tan Z., Xuan Z., Wu C., Cheng Y., Xu C., Ma X., Wang D. (2022). Effects of selenium on the AsA-GSH system and photosynthesis of pakchoi (*Brassica chinensis* L.) under lead stress. J. Soil Sci. Plant Nutr..

[6] Obroucheva N., Bystrova E., Ivanov V., Antipova O., Seregin I. (1998). Root growth responses to lead in young maize seedlings. Plant Soil.

[7] Ji Y., Ren Y., Han C., Zhu W., Gu J., He J. (2022). Application of exogenous glycinebetaine alleviates lead toxicity in pakchoi (*Brassica chinensis* L.) by promoting antioxidant enzymes and suppressing Pb accumulation. Environ. Sci. Pollut. Res..

[8] Li T., Li B., Wang Y., Xu J., Li W., Chen Z.H., Mou W., Xue D. (2025). WRKY Transcription Factors in Rice: Key Regulators Orchestrating Development and Stress Resilience. Plant Cell Environ..

[9] Eulgem T., Rushton P.J., Robatzek S., Somssich I.E. (2000). The WRKY superfamily of plant transcription factors. Trends Plant Sci..

[10] Chen F., Hu Y., Vannozzi A., Wu K., Cai H., Qin Y., Mullis A., Lin Z., Zhang L. (2017). The WRKY transcription factor family in model plants and crops. Crit. Rev. Plant Sci..

[11] Ma Z., Hu L. (2024). WRKY transcription factor responses and tolerance to abiotic stresses in plants. Int. J. Mol. Sci..

[12] Wang H., Chen W., Xu Z., Chen M., Yu D. (2023). Functions of WRKYs in plant growth and development. Trends Plant Sci..

[13] Lippok B., Birkenbihl R.P., Rivory G., Brümmer J., Schmelzer E., Logemann E., Somssich I.E. (2007). Expression of AtWRKY33 encoding a pathogen-or PAMP-responsive WRKY transcription factor is regulated by a composite DNA motif containing W box elements. Mol. Plant-Microbe Interact..

[14] Fang X., Meng X., Zhang J., Xia M., Cao S., Tang X., Fan T. (2021). AtWRKY1 negatively regulates the response of Arabidopsis thaliana to Pst. DC3000. Plant Physiol. Biochem..

[15] Ding Z.J., Yan J.Y., Li C.X., Li G.X., Wu Y.R., Zheng S.J. (2015). Transcription factor WRKY 46 modulates the development of Arabidopsis lateral roots in osmotic/salt stress conditions via regulation of ABA signaling and auxin homeostasis. Plant J..

[16] Zhang M., Zhao R., Huang K., Huang S., Wang H., Wei Z., Li Z., Bian M., Jiang W., Wu T. (2022). The OsWRKY63–OsWRKY76–OsDREB1B module regulates chilling tolerance in rice. Plant J..

[17] Yan J., Li J., Zhang H., Liu Y., Zhang A. (2022). ZmWRKY104 positively regulates salt tolerance by modulating ZmSOD4 expression in maize. Crop J..

[18] Sheng Y., Yan X., Huang Y., Han Y., Zhang C., Ren Y., Fan T., Xiao F., Liu Y., Cao S. (2019). The WRKY transcription factor, WRKY13, activates PDR8 expression to positively regulate cadmium tolerance in Arabidopsis. Plant Cell Environ..

[19] Gu L., Hou Y., Sun Y., Chen X., Wang G., Wang H., Zhu B., Du X. (2024). The maize WRKY transcription factor ZmWRKY64 confers cadmium tolerance in Arabidopsis and maize (*Zea mays* L.). Plant Cell Rep..

[20] Hong C., Cheng D., Zhang G., Zhu D., Chen Y., Tan M. (2017). The role of ZmWRKY4 in regulating maize antioxidant defense under cadmium stress. Biochem. Biophys. Res. Commun..

[21] Li G.Z., Wang Z.Q., Yokosho K., Ding B., Fan W., Gong Q.Q., Li G.X., Wu Y.R., Yang J.L., Ma J.F. (2018). Transcription factor WRKY 22 promotes aluminum tolerance via activation of Os FRDL 4 expression and enhancement of citrate secretion in rice (*Oryza sativa*). New Phytol..

[22] He S., An R., Yan J., Zhang C., Zhang N., Xi N., Yu H., Zou C., Gao S., Yuan G. (2023). Association studies of genes in a Pb response-associated network in maize (*Zea mays* L.) reveal that ZmPIP2; 5 is involved in Pb tolerance. Plant Physiol. Biochem..

[23] Hou F., Liang Y., Sang M., Zhao G., Song J., Liu P., Zou C., Chen Z., Ma L., Shen Y. (2025). Complex regulatory network of ZmbZIP54-mediated Pb tolerance in maize. Plant Physiol. Biochem..

[24] Li Z., Jiang L., Wang C., Liu P., Ma L., Zou C., Pan G., Shen Y. (2023). Combined genome-wide association study and gene co-expression network analysis identified ZmAKINβγ1 involved in lead tolerance and accumulation in maize seedlings. Int. J. Biol. Macromol..

[25] Hou F., Zhang N., Ma L., An L., Zhou X., Zou C., Yang C., Pan G., Lübberstedt T., Shen Y. (2023). ZmbZIP54 and ZmFDX5 cooperatively regulate maize seedling tolerance to lead by mediating ZmPRP1 transcription. Int. J. Biol. Macromol..

[26] Pan Y., Han T., Xiang Y., Wang C., Zhang A. (2024). The transcription factor ZmNAC84 increases maize salt tolerance by regulating ZmCAT1 expression. Crop J..

[27] Jia Z., Li M., Wang H., Zhu B., Gu L., Du X., Ren M. (2021). TaWRKY70 positively regulates TaCAT5 enhanced Cd tolerance in transgenic Arabidopsis. Environ. Exp. Bot..

[28] Zhang X., Ma Y., Zhang W., Ji M., Dong J., Lai D., Yu W., Zhang X., Zhu Y., Wang Y. (2025). RsWRKY75 promotes ROS scavenging and cadmium efflux via activating the transcription of RsAPX1 and RsPDR8 in radish (*Raphanus sativus* L.). Plant Cell Rep..

[29] Han Z., Wang J., Wang X., Zhang X., Cheng Y., Cai Z., Nian H., Ma Q. (2022). GmWRKY21, a soybean WRKY transcription factor gene, enhances the tolerance to aluminum stress in Arabidopsis thaliana. Front. Plant Sci..

[30] Dang F., Lin J., Chen Y., Li G.X., Guan D., Zheng S.J., He S. (2019). A feedback loop between CaWRKY41 and H_2_O_2_ coordinates the response to Ralstonia solanacearum and excess cadmium in pepper. J. Exp. Bot..

[31] Zhou L., John Martin J.J., Li R., Zeng X., Wu Q., Li Q., Fu D., Li X., Liu X., Ye J. (2024). Catalase (CAT) Gene Family in Oil Palm (*Elaeis guineensis* Jacq.): Genome-Wide Identification, Analysis, and Expression Profile in Response to Abiotic Stress. Int. J. Mol. Sci..

[32] Hu L., Yang Y., Jiang L., Liu S. (2016). The catalase gene family in cucumber: Genome-wide identification and organization. Genet. Mol. Biol..

[33] Jiang W., Ye Q., Wu Z., Zhang Q., Wang L., Liu J., Hu X., Guo D., Wang X., Zhang Z. (2023). Analysis of CAT gene family and functional identification of OsCAT3 in rice. Genes.

[34] Su T., Wang P., Li H., Zhao Y., Lu Y., Dai P., Ren T., Wang X., Li X., Shao Q. (2018). The Arabidopsis catalase triple mutant reveals important roles of catalases and peroxisome-derived signaling in plant development. J. Integr. Plant Biol..

[35] Willekens H., Villarroel R., Van Montagu M., Inzé D., Van Camp W. (1994). Molecular identification of catalases from *Nicotiana plumbaginifolia* (L.). FEBS Lett..

[36] Tounsi S., Kamoun Y., Feki K., Jemli S., Saïdi M.N., Ziadi H., Alcon C., Brini F. (2019). Localization and expression analysis of a novel catalase from Triticum monococcum TmCAT1 involved in response to different environmental stresses. Plant Physiol. Biochem..

[37] Liu Z., Wang D., Tang H., Li H., Zhang X., Dong S., Zhang L., Yang L. (2023). Identification and analysis of the catalase gene family response to abiotic stress in *Nicotiana tabacum* L.. Agronomy.

[38] Mhamdi A., Queval G., Chaouch S., Vanderauwera S., Van Breusegem F., Noctor G. (2010). Catalase function in plants: A focus on Arabidopsis mutants as stress-mimic models. J. Exp. Bot..

[39] Du Y.Y., Wang P.C., Chen J., Song C.P. (2008). Comprehensive functional analysis of the catalase gene family in *Arabidopsis thaliana*. J. Integr. Plant Biol..

[40] Jiang Y., Liang G., Yang S., Yu D. (2014). Arabidopsis WRKY57 functions as a node of convergence for jasmonic acid–and auxin-mediated signaling in jasmonic acid–induced leaf senescence. Plant Cell.

[41] Han Y., Fan T., Zhu X., Wu X., Ouyang J., Jiang L., Cao S. (2019). WRKY12 represses GSH1 expression to negatively regulate cadmium tolerance in *Arabidopsis*. Plant Mol. Biol..

[42] Liu Y., Wang S., Liu X., Wang R., Chen W., Suo J., Yan J., Wu J. (2024). Agrobacterium-mediated transient expression in Torreya grandis cones: A simple and rapid tool for gene expression and functional gene assay. Sci. Hortic..

[43] Han T., Wang L., Jiang Q., Wang M., Wang C., Wang M., Zhao J., Tian S., Liu C., Yang Y. (2024). Extracellular adenosine triphosphate (eATP) alleviates phytotoxicity of nanoplastics in maize (*Zea mays* L.) plants. Environ. Exp. Bot..

[44] Li Q., Wang M., Fang L. (2021). BASIC PENTACYSTEINE2 negatively regulates osmotic stress tolerance by modulating LEA4-5 expression in Arabidopsis thaliana. Plant Physiol. Biochem..

[45] Meng L., Yang Y., Ma Z., Jiang J., Zhang X., Chen Z., Cui G., Yin X. (2022). Integrated physiological, transcriptomic and metabolomic analysis of the response of Trifolium pratense L. to Pb toxicity. J. Hazard. Mater..

[46] Liu Z., Yan J., Chen J., Wang T., Li X., Wang S., Wang R., Liu Y., Yan J., Wu J. (2025). The TgIAA7-TgARF10-TgProDH1 module regulates waterlogging-mediated proline accumulation in the gymnosperm Torreya grandis. Plant Physiol..

[47] Yan J., Liu Z., Wang T., Wang R., Wang S., Liu X., Liu Y., Yan J., Wu J. (2025). TgRAV1–TgWRKY74–TgACS13 module fine-tunes ethylene biosynthesis to modulate waterlogging-induced root vitality in the gymnosperm Torreya grandis. J. Integr. Plant Biol..

