# Supplementary figures and images for: The Maize WRKY Transcription Factor ZmWRKY4 Confers Lead Tolerance by Regulating ZmCAT1 Expression
Source: Plants (Basel). 2026 Jan 28;15(3):394. doi: 10.3390/plants15030394 (PMC12899097; doi:10.3390/plants15030394)

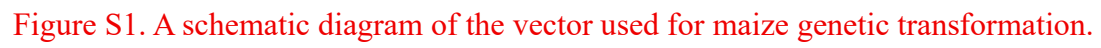

Figure S1. A schematic diagram of the vector used for maize genetic transformation.

Supplement: Supplementary file 1 [file plants-15-00394-s001.zip › Supplemental Figures.pdf]
